# Apolipoprotein E ε4 Specifically Modulates the Hippocampus Functional Connectivity Network in Patients With Amnestic Mild Cognitive Impairment

**DOI:** 10.3389/fnagi.2018.00289

**Published:** 2018-09-27

**Authors:** Lin Zhu, Hao Shu, Duan Liu, Qihao Guo, Zan Wang, Zhijun Zhang

**Affiliations:** ^1^Department of Neurology, Affiliated ZhongDa Hospital, School of Medicine, Southeast University, Nanjing, China; ^2^Department of Neurology, Huashan Hospital, Fudan University, Shanghai, China

**Keywords:** Alzheimer’s disease (AD), amnestic mild cognitive impairment (aMCI), apolipoprotein E (APOE) ε4, resting-state fMRI, functional connectivity

## Abstract

The presence of both apolipoprotein E (APOE) ε4 allele and amnestic mild cognitive impairment (aMCI) are considered to be risk factors for Alzheimer’s disease (AD). Numerous neuroimaging studies have suggested that the modulation of APOE ε4 affects intrinsic functional brain networks, both in healthy populations and in AD patients. However, it remains largely unclear whether and how ε4 allele modulates the brain’s functional network architecture in subjects with aMCI. Using resting-state functional magnetic resonance imaging (fMRI) and graph-theory approaches-functional connectivity strength (FCS), we investigate the topological organization of the whole-brain functional network in 28 aMCI ε4 carriers and 38 aMCI ε3ε3 carriers. In the present study, we first observe that ε4-related FCS increases in the right hippocampus/parahippocampal gyrus (HIP/PHG). Subsequent seed-based resting-state functional connectivity (RSFC) analysis revealed that, compared with the ε3ε3 carriers, the ε4 carriers had lower or higher RSFCs between the right HIP/PHG seed and the bilateral medial prefrontal cortex (MPFC) or the occipital cortex, respectively. Further correlation analyses have revealed that the FCS values in the right HIP/PHG and lower HIP/PHG-RSFCs with the bilateral MPFC were significantly correlated with the impairment of episodic memory and executive function in the aMCI ε4 carriers. Importantly, the logistic regression analysis showed that the HIP/PHG-RSFC with the bilateral MPFC predicted aMCI-conversion to AD. These findings suggest that the APOE ε4 allele may modulate the large-scale brain network in aMCI subjects, facilitating our understanding of how the entire assembly of the brain network reorganizes in response to APOE variants in aMCI. Further longitudinal studies need to be conducted, in order to examine whether these network measures could serve as primary predictors of conversion from aMCI ε4 carriers to AD.

## Introduction

Alzheimer’s disease (AD) is one of the most common neurodegenerative diseases in the world and is traditionally defined as a type of dementia. In order to predict the conversion of individuals with suspected AD, mild cognitive impairment (MCI) has been extended as an early stage of dementia in the clinical spectrum (Petersen, 2016; De Belder et al., 2017). Amnestic mild cognitive impairment (aMCI) is generally regarded as a transitional condition between the normal cognition and very early dementia, based on age and educational level (Camarda et al., 2018; Giulietti et al., 2018). This is the pivotal time to recognize aMCI from aging, to delay the progression of AD. However, the prognosis of aMCI is highly variable, which means that some patients with aMCI will progress to dementia, while some will remain stable, and some will even revert to being normal. Therefore, it is essential for patients with aMCI to have early diagnosis and precise individual therapy.

The onset of AD is influenced by several distinct factors, including physiological, environmental and genetic factors (Petersen, 2000; Buckner, 2004). The apolipoprotein E (APOE) gene consists of polymorphic ε2, ε3 and ε4 alleles, with worldwide frequencies of 8.4, 77.9 and 13.7%, respectively, in cognitive normal subjects; AD patients show a 40% increase in ε4 allele frequency (Farrer et al., 1997) with gender independence (Neu et al., 2017). Nearly identical changes in the pattern of functional magnetic resonance imaging (fMRI) activity during memory tasks were shown in ε2 and ε4 carriers (Trachtenberg et al., 2012a,b; Shu et al., 2016), thereby challenging the protective effect of ε2. On the other hand, APOE ε4 allele is an acknowledged genetic factor of vulnerability for AD patients with dose-dependent effects on clinical phenotype (Spinney, 2014). Inheritance of an APOE ε4 allele would greatly increase the risk of AD (up to threefold). Moreover, two APOEε4 alleles can cause a 12-fold increase in the risk of AD (Karch and Goate, 2015). Also, APOE ε4 carriers have been demonstrated to experience an earlier onset of memory decline and to have greater rates of disease progression than non-APOE ε4 carriers (Ungar et al., 2014). Neuropsychological studies have revealed that APOE ε4 has a significant effect on general cognitive function (Tangwongchai et al., 2018), especially episodic memory (Rajah et al., 2017). Neuropathological studies in human and animal models have proved that APOE ε4 has a physical interaction with Aβ, which has a strong influence on memory deficit in probable preclinical AD (Holtzman et al., 2012). In addition, the interaction is closely associated with memory decline at each age and will be exacerbated with aging (Lim et al., 2018). Neuroimaging studies demonstrate that APOE genotypes have remarkable influences on both gray matter volume (Chen et al., 2015) and white matter volume (Wang et al., 2017b) in subjects with aMCI. The cortex atrophy of hippocampus, amygdala and anterior cingulate have been found in stable MCI patients with APOE ε4 (Hämäläinen et al., 2008; Liu et al., 2010; Tang et al., 2015), and this will extend to frontal and partial and temporal lobe in AD (Hämäläinen et al., 2008). Furthermore, a smaller hippocampus size is significantly correlated with lower plasma APOE levels (Teng et al., 2015). Numerous fMRI studies report abnormal regional brain activation as well as large-scale brain networks in MCI and AD. On memory tasks, AD patients show hypoactivation in regions of the hippocampus and middle temporal areas compared to aMCI patients and healthy controls (Petrella et al., 2007), whereas MCI patients show hyperactivation in hippocampal areas, fusiform gyrus, anterior cingulate gyrus, frontal and temporal regions relative to heathy controls (Yetkin et al., 2006; Trivedi et al., 2008). On the other hand, resting-state fMRI (rfMRI) studies show reduced connectivity between medial temporal structures and the posterior cingulate cortex in AD (Greicius et al., 2004) and increased connectivity within medial temporal lobe in MCI. Additionally, APOE ε4 have greatly influence on the alteration of functional connectivity. Seed-based analysis indicate increased connectivity between the prefrontal/parietal/temporal cortex and the hippocampus (De Marco et al., 2017b; Zheng et al., 2018) in APOE ε4 carriers of both young healthy adults and MCI patients. In AD, APOE ε4 can profoundly disrupted whole-brain topological organization as well (Wang et al., 2015a). However, it remains unclear whether and how APOE ε4 allele modulates the brain’s functional network architecture in subjects with aMCI. Therefore, rfMRI and graph-theory approaches are used to investigate the APOE ε4 effects on topological organization of the whole-brain functional network.

In the present study, 66 aMCI subjects, including 28 ε4 carriers and 38 ε3ε3 carriers, were recruited. The study uses R-fMRI to investigate the whole-brain functional connectivity patterns in aMCI ε4 carriers and ε3ε3 carriers. The whole-brain functional networks are constructed by measuring the temporal correlations of every pair of voxels in the brain and are analyzed by using the graph-theory approaches. We hypothesize that APOE ε4 allele may profoundly affect the functional disintegration of brain networks with a specific pattern in aMCI patients.

## Materials and Methods

### Participants

The current study recruited a group of 66 aMCI subjects, consisting of 28 ε4 carriers and 38 ε3ε3 carriers, from the Nanjing Aging and Dementia Study (NADS) dataset (Shu et al., 2018). All the subjects are Chinese Han and right-handed. Written informed consent has been provided by all participants, and study protocols have been approved in accordance with the Human Participants Ethics Committee of the Affiliated ZhongDa Hospital, Southeast University. All the subjects have submitted their personal information and have received neuropsychological and comprehensive neurological assessments, and APOE genotyping.

### Clinical Evaluation

All participants were assessed for general cognitive function by using the Mini-Mental State Examination (MMSE) and Mattis Dementia Rating Scale-2 (MDRS-2). Neuropsychological tests were used to assess different cognitive domains, which include episodic memory (three tests, including the auditory verbal learning test with a 20-min delayed recall (AVLT-20 min DR), the logical memory test with a 20-min delayed recall (LMT-20 min DR), and the Rey-Osterrieth complex figure test with a 20-min DR (CFT-20 min DR), visuospatial function (two tests, including the Rey-Osterrieth complex figure test (CFT) and the clock drawing test (CDT)), information processing speed (four tests, including the digital symbol substitution test (DSST), the trail making test A (TMT-A), and Stroop color-word test A and B (Stroop A and B)), and executive function (five tests, including the verbal fluency test (VFT), the digital span test-backward (DST-backward), the trail making test-B (TMT-B), Stroop color-word test C (Stroop C) and the semantic similarity test (similarity).

In this study, we performed a composite score analysis of these cognitive function measures to increase statistical power by reducing random variability and floor and ceiling effects (Shu et al., 2016). In brief, for each subject, the raw scores from each test were first transformed to z scores, with reference to the means and standard deviation (SD)s of the test. Then, the composite scores were performed by averaging the z scores of the individual tests related to the four cognitive domains. Notably, MMSE and MDRS-2 were used for descriptive and diagnostic classifications, but not for the composite measures.

### Inclusion and Exclusion Criteria

The diagnostic criteria proposed by Petersen (2004) was met for all aMCI subjects, as well as the other following criteria: (1) age was between 60 and 80; (2) right-handed; (3) education level was above junior middle school; (4) have adequate acuity in audition and vision for the neuropsychological assessment; (5) subjective memory impairment corroborated by the subject and an informant; (6) objective memory performance documented by the AVLT-20-min DR score of less than or equal to 1.5 times the SD of age-adjusted and education-adjusted norms (the cutoff was ≤4 correct responses on 12 items for ≥8 years of education); (7) normal general cognitive function evaluated by MMSE score ≥24 or MDRS-2 ≥120; (8) preserved activities of daily living; (9) insufficient level to meet the Alzheimer’s Criteria of National Institute of Neurological and Communicative Disorders and Stroke and the AD and Related Disorders Association (NINCDS-ADRDA). Exclusion criteria included: (1) history of explicit diagnosis of neurological or psychiatric diseases, such as stoke, Parkinson’s disease, epilepsy, head injury, depression; (2) contraindication for MRI scans and not tolerant to MRI scans; (3) severe visual or hearing loss; (4) gross structural abnormalities on T1-weighted images, and major white matter changes, such as infarction or other vascular lesions on T2-weighted MRI.

### APOE Genotyping

The genomic deoxyribonucleic acid (DNA) of each subject was extracted by a DNA extraction kit (Tiangen, China) from 250 μl ethylene diamine tetra acetic (EDTA) anticoagulation, making use of a polymerase chain reaction-based restriction fragment length polymorphism (PCR-RFLP) assay to detect rs7412 and rs429358 alleles, respectively. Also, the haplotype of rs7412 and rs429358 makes the determination of the APOE genotype.

### MRI Data Acquisition

All MRI scans were performed in a 3.0-T Siemens Verio scanner (Siemens, Erlangen, Germany) with a 12-channel head coil at the Affiliated ZhongDa Hospital of Southeast University. During scanning, the subjects wearing earplugs and whose heads were immobilized by a pair of stabilizers were required to lie still and close their eyes. First, high-resolution T1-weighted axial images were obtained to cover the whole brain with a 3D magnetization prepared rapid gradient echo (MPRAGE) sequence, as follows: repetition time (TR)/echo time (TE) = 1,900/2.48 ms; acquired matrix = 256 × 256; flip angle (FA) = 9°; number of slices = 176; field of view (FOV) = 250 × 250 mm^2^, thickness = 1.0 mm; gap = 0 mm. The RfMRIs were acquired for 8 min through a gradient-recalled echo-planar imaging (GRE-EPI) sequence: TR/TE = 2,000/25 ms; acquisition matrix = 64 × 64; FA = 90°; number of slices = 36; FOV = 250 × 250 mm^2^; thickness = 4.0 mm; gap = 0 mm. Moreover, axial T2-weighted images were acquired, in order to exclude subjects who had cerebral infraction, severe white matter changes or other obvious lesions.

### MRI Data Preprocessing

Statistical Parametric Mapping (SPM8)^1^ and the Data Processing Assistant for Resting-State fMRI (DPARSF)^2^ were performed to make data preprocessing. The first 10 functional volumes were discarded, and the remaining images were corrected for timing differences and motion effects. Next, all participants with 3° of angular motion or head motion >3 mm maximum displacement in the *x*, *y*, or *z* direction were excluded. The resulting images were spatially normalized into the standard Montreal Neurological Institute (MNI) EPI template using the default settings, resampled to 3 × 3 × 3 mm^3^ voxels. Then, linear de-trending and temporal band-pass filtering (0.01–0.1 Hz) were conducted, in order to reduce the effects of high-frequency physiological noise, as well as low-frequency drift.

### Network Analysis

The whole-brain network functional connectivity analysis was performed with the application of GRETNA package^3^ (Wang et al., 2015b). In brief, for each subject, a whole-brain functional connectivity matrix was generated among all subjects by computing the Pearson’s correlations between the time series of all pairs of brain voxels with a gray matter mask (*N* voxels = 57,641) application and a threshold of 0.2 setting on the mean gray matter probability map. Furthermore, in order to quantify the functional integrity of the whole-brain network, functional connectivity strength (FCS) analysis was performed (Wang et al., 2014).

### Statistical Analysis

#### Demographic and Neuropsychological Variables

For gender assessment, *χ*^2^ tests were used. The age and education level differences between the two groups were estimated by an independent samples *t*-test. In addition, one-way analysis of covariance (ANCOVA) was carried out to assess cognitive performance, with age, gender and years of education as covariates. These analyses were implemented in SPSS 18.0 (SPSS Inc., Chicago, IL, USA).

#### Group Differences in Functional Connectivity Strength (FCS)

To analyze the whole-brain functional connectivity, first we performed the Pearson’s correlations between the time series of all pairs of voxels for each participant, constructing a whole-brain functional connectivity matrix. In addition, this computation was constrained within a gray matter mask (*N_voxels_* = 57,641), which was generated by setting a threshold of 0.2 on the mean map of all gray matter across all subjects. In order to improve normality, a Fisher’s r-to-z transformation was performed, in order to transform individual correlation matrices to a single z-score matrix. Second, all individual FCS maps were spatially smoothed with a Gaussian kernel (full width at half-maximum = 6 mm). Last, a voxel-wise one-way ANCOVA was performed to examine the between-group (aMCI ε4 carriers vs. ε3ε3 carriers) differences in FCS maps with age, gender and years of education as covariates. In addition, Monte Carlo simulations were conducted for correction in multiple comparisons using the AlphaSim program^4^. The α level of 0.05 was obtained with a voxel-wise *P* < 0.05 and cluster size >4,266 mm^3^.

#### Seed-Based Resting-State Functional Connectivity Analysis

To further examine the detailed resting-state functional connectivity (RSFC) alterations, we performed a seed-based connectivity analysis, using the clusters showing significant between-group differences in FCS as the seeds. The one-way ANCOVA was performed on the RSFC maps for the identified seed (i.e., right hippocampus/parahippocampal gyrus (HIP/PHG), see “Results” section). The significant level was set at *P* < 0.05 with a cluster size of 82 voxels, corresponding to a corrected *P* < 0.05. The analysis mask was generated by selecting the voxels showing significant positive RSFC in the two groups.

#### Relationships Between Regional Connectivity Measures and Cognitive Performance

Multiple linear regression analyses were performed to examine the associations between the cognitive measures and the connectivity measurements (i.e., FCS and seed-based RSFC) in the areas showing significant between-group differences, with age, gender and years of education as covariates. To statistically compute the differences in magnitudes of the correlations between the reginal functional connectivity and cognitive performance, the correlation coefficients obtained above were further converted into z values by using Fisher’s r-to-z transform. A *Z* statistic was then performed to compare these transformed z values, in order to determine the significance of the between-group differences in correlations. In addition, Cohen’s *q* was used to quantify the magnitude of difference between correlations; |Cohen’s *q*| <0.20 was considered a small effect, 0.30 a moderate effect, and 0.50 a large effect (Lim et al., 2013).

## Results

### Demographic Information and Neuropsychological Characteristics for All Subjects

The demographic information and neuropsychological characteristics of all subjects are shown in Table 1. Compared with the APOE ε3ε3 carriers, the APOE ε4 carriers showed no significant differences in age, gender, education level and cognition performances (all *P*s >0.05).

**Table 1:** Demographic and neuropsychological data for all subjects.

	aMCI		*P* value	Cohen’s *d*
	APOE ε3ε3 (*n* = 38)	APOE ε4+ (*n* = 28)		
**Percentage female**	17 (44.74%)	13 (46.43%)	0.891^a^	−
**Age** (years)	69.68 ± 7.17	71.5 ± 6.81	0.303^b^	−
**Education level** (category)	7–18 years	8–19.5 years	0.869^b^	−
**General cognition**				
MMSE	26.08 ± 2.65	25.71 ± 3.10	0.963^c^	0.13
MDRS-2 total	130.82 ± 6.92	129.50 ± 6.86	0.746^c^	0.19
**Composite Z scores of each cognition domain**				
Episodic memory	0.13 ± 0.82	−0.18 ± 0.87	0.259^c^	0.37
AVLT-20 min DR	0.11 ± 1.03	−0.14 ± 0.96	0.545^c^	0.25
LMT-20 min DR	0.09 ± 0.98	−0.12 ± 1.04	0.540^c^	0.21
CFT-20 min DR	0.21 ± 1.01	−0.28 ± 0.93	0.101^c^	0.51
Visuospatial function	0.10 ± 0.80	−0.13 ± 0.51	0.338^c^	0.34
CDT	0.09 ± 1.02	−0.12 ± 0.98	0.685^c^	0.21
CFT	0.11 ± 1.31	−0.14 ± 0.08	0.382^c^	0.25
Information processing speed	0.02 ± 0.79	−0.02 ± 0.82	0.638^c^	0.05
DSST	0.04 ± 0.97	−0.05 ± 1.06	0.810^c^	0.09
TMT-A	0.04 ± 0.95	−0.05 ± 1.07	0.926^c^	0.09
Stroop A	0.01 ± 1.08	−0.02 ± 0.90	0.613^c^	0.03
Stroop B	−0.02 ± 1.09	0.03 ± 0.88	0.556^c^	−0.05
Executive function	0.02 ± 0.55	−0.03 ± 0.68	0.747^c^	0.08
VFT-objects	0.02 ± 0.99	−0.02 ± 1.03	0.870^c^	0.04
VFT-animals	0.06 ± 0.92	−0.08 ± 1.11	0.930^c^	0.14
DST-backward	−0.05 ± 0.97	0.06 ± 1.06	0.549^c^	−0.11
TMT-B	0.10 ± 0.97	−0.13 ± 1.04	0.745^c^	0.23
Stroop C	−0.12 ± 1.04	0.17 ± 0.93	0.096^c^	−0.30
Similarity	0.13 ± 0.94	−0.18 ± 1.06	0.331^c^	0.32

*Data are presented as mean ± standard deviation. The level of each cognitive domain and performance of each neuropsychological test are expressed as Z scores. aMCI, amnestic mild cognitive impairment; APOE, apolipoprotein E; MMSE, Mini-Mental State Exam; MDRS-2, Mattis dementia rating scale-2. AVLT, auditory verbal learning test; LMT, logical memory test; CFT, Rey-Osterrieth complex figure test; CDT, clock drawing test; CFT, Rey-Osterrieth complex figure test; DSST, digital symbol substitution test; TMT-A, trail making test-A; Stroop, Stroop color test; VFT, verbal fluency test; DST, digit span test; TMT-B, trail making test-B; Similarity, semantic similarity test. ^a^P values were obtained by χ2 tests. ^b^P values were obtained by independent samples t-test. ^c^P values were obtained by one-way analysis of covariance (ANCOVA)*.

### Between-Group Differences in FCS Maps

**Figure 1:**
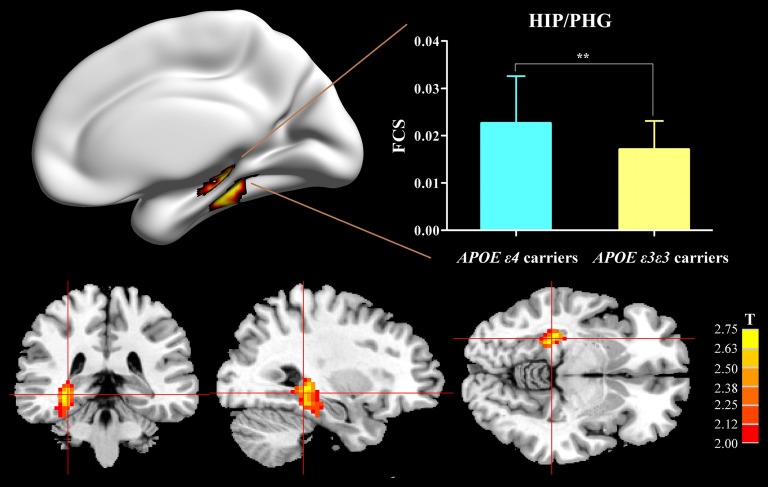
Difference of FCS map between APOE ε3ε3 carriers and APOE ε4+ carriers. Compared with the APOE ε3ε3 carriers, the APOE ε4+ carriers showed significantly increased FCS values in the right hippocampus/parahippocampus. APOE, apolipoprotein E; HIP, hippocampus; PHG, parahippocampal gyrus; FCS, functional connectivity strength; **P < 0.01.

Compared with the APOE ε3ε3 carriers, the APOE ε4 carriers displayed increased FCS, primarily in the HIP/PHG (Figure 1).

### APOE ε4-Related Alterations in Seed-Based RSFC Maps

For further detailed analysis regarding the right HIP/PHG networks in aMCI ε3ε3 carriers and ε4 carriers, the subsequent seed-based RSFC analysis revealed that the RSFC patterns were largely similar across the APOE ε3ε3 and APOE ε4 groups (Figure 3). Each hippocampus sub-regional network was composed of diffuse subcortical, medial frontal, temporal cortical, parietal and cerebellar sites. The RSFC pattern was similar to that in previous studies with the hippocampus as the seed region. However, compared with the ε3ε3 carriers, the ε4 carriers had lower RSFCs between the right HIP/PHG seed and the bilateral MPFC, but higher RSFCs between the right HIP/PHG seed and the occipital cortex (i.e., left middle occipital gyrus, right lingual gyrus and inferior occipital gyrus; Figure 4A and Table 3).

**Figure 2:**
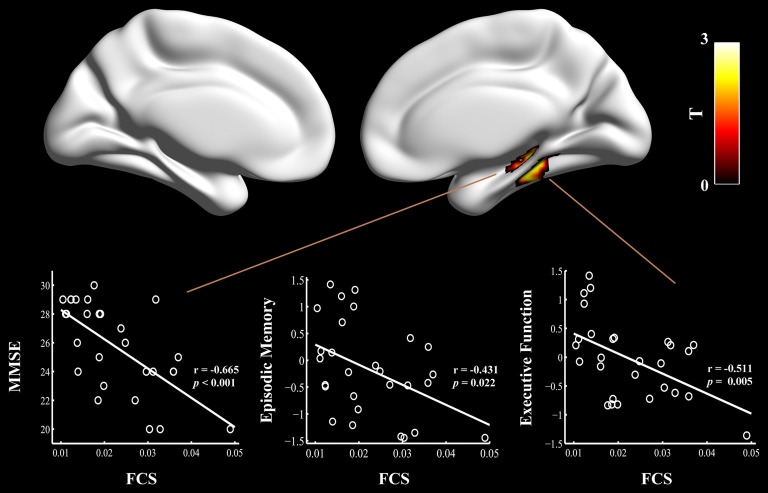
Relationship between cognitive performances and regional FCS values in the aMCI patients carrying APOE ε4. The scatter plots show correlations between cognitive performance (e.g., episodic memory and executive function) and regional FCS values in the right hippocampus/para-hippocampal gyrus in the aMCI ε4 carriers. FCS, functional connectivity strength; aMCI, amnestic mild cognitive impairment; APOE, apolipoprotein E; MMSE, Mini-Mental State Exam.

**Figure 3:**
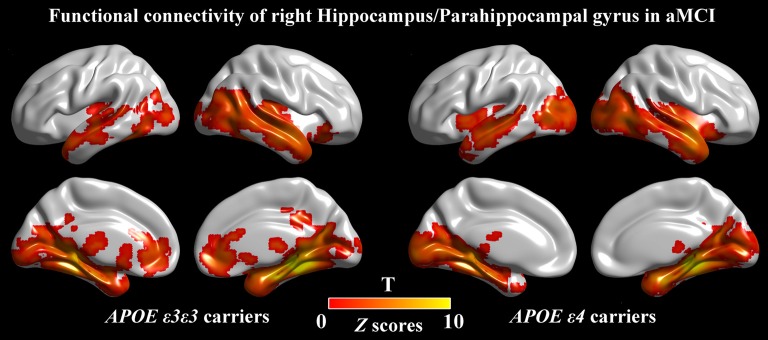
Seed-based RSFC maps. For the seed region, the RSFC patterns were largely similar across the APOE ε3ε3 and APOE ε4 groups. The color bar represents the strength of positive RSFC. APOE, apolipoprotein E; RSFC, resting-state functional connectivity.

**Table 2:** Correlations between regional FCS values and cognitive scores in aMCI.

	r, APOE ε3ε3 carriers (*n* = 38)	r, APOE ε4+carriers (*n* = 28)	Fisher’s *Z*	Cohen’s *q*
**MMSE**	**−0.165**	−0.655**	**2.36 (0.0092)**	**0.6175**
**Episodic memory**	−0.221	−0.431*	0.90 (0.1833)	0.2364
**Visuospatial function**	−0.045	−0.366	1.29 (0.0979)	0.3388
**Information processing speed**	−0.039	−0.338	1.19 (0.1161)	0.3128
**Executive function**	−0.054	−0.511**	1.95 (0.0257)	**0.5100**

Multiple linear regression analyses were conducted to calculate the correlations between the regional FCS values and cognitive performance in the APOE ε3ε3 and APOE ε4 carriers, after controlling for age, gender and education. To statistically compute the differences in magnitudes of the correlations between the regional FCS values and cognitive performance in the APOE ε3ε3 and APOE ε4 carriers, correlation coefficients obtained above were further converted into z values by using Fisher’s r-to-z transform. A Z statistic was then used to compare these transformed z values to determine the significances of the between-group differences in correlations, and Cohen’s q was used to quantify the magnitude of difference between correlations; according to |Cohen’s q| < 0.20 is considered a small effect, 0.30 a moderate effect, and 0.50 a large effect. aMCI, amnestic mild cognitive impairment; APOE, apolipoprotein E; FCS, functional connectivity strength *P < 0.05 **P < 0.01. Values in bold means that compared with HC group, these values make significant correlation with reginal FCS in aMCI patients.

**Table 3:** Regions showing altered functional connectivity for APOE ε4 carriers and APOE ε3ε3 carriers using the right HIP/PHG seed (*x* = 30 mm, *y* = −36 mm, *z* = −6 mm).

Brain regions	MNI coordinates (mm)			Clusters sizes (voxels)
	*x*	*y*	*z*	
**APOE ε4+ carriers < APOE ε3ε3 carriers**
Bilateral MPFC	24	27	−18	178
**APOE ε4+ carriers >APOE ε3ε3 carriers**
Left middle occipital gyrus	−24	−87	6	112
Right lingual gyrus/inferior occipital gyrus	33	−90	−6	83

*APOE ε4-related alterations in the RSFC between the right HIP/PHG and bilateral MPFC, left middle occipital gyrus and right lingual gyrus/inferior occipital gyrus were observed, with significant lower values in bilateral MPFC and increased values in the left middle occipital gyrus and right lingual gyrus/inferior occipital gyrus in the APOE ε4 carriers compared to the APOE ε3ε3 carriers. APOE, apolipoprotein E; MPFC, medial prefrontal cortex; HIP, hippocampus; PHG, parahippocampal gyrus; RSFC, resting-state functional connectivity*.

**Figure 4:**
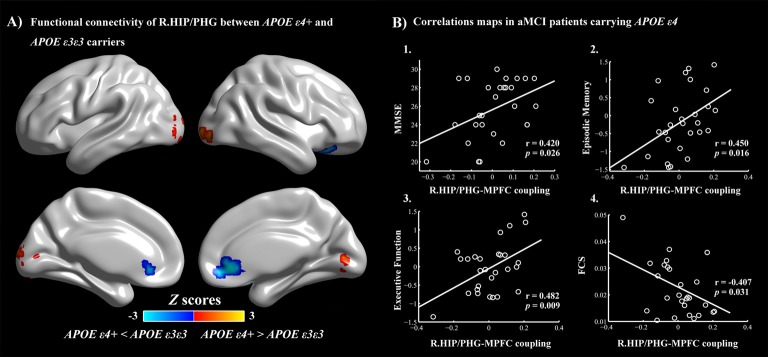
**(A)** APOE ε4-related alterations in the HIP/PHG-RSFC. Red: APOE ε4+ >APOE ε3ε3 (left middle occipital gyrus, right lingual gyrus and inferior occipital gyrus). Blue: APOE ε4+ <APOE ε3ε3 (bilateral MPFC). The scatter maps show significant correlations between the HIP/PHG-RSFC with the bilateral MPFC and the cognitive performances **(B1–B3)**. Significant correlation between the HIP/PHG-RSFC with the bilateral MPFC and regional FCS values in the right HIP/PHG was also observed in the aMCI ε4 carriers **(B4)**. APOE, apolipoprotein E; RSFC, resting-state functional connectivity; HIP, hippocampus; PHG, parahippocampal gyrus; MPFC, medial prefrontal cortex; FCS, functional connectivity strength.

### Logistic Regression Analysis for Conversion From aMCI to AD

In this dataset, 43 aMCI patients received, on average, a 3-year follow-up after baseline, and 13 aMCI patients had converted to AD. A further multivariate logistic model was used to test the prognostic accuracy of the FCS, seed-based RSFC values and APOE genotype status for aMCI-conversion prediction. We found that the HIP/PHG-RSFC with the bilateral MPFC predicted aMCI-conversion to AD (95% CI 0.000–0.768; *P* = 0.042).

### Correlations Between Cognitive Performances and Connectivity Measures

Finally, multivariate regression analyses were performed to examine the associations between the cognitive performances (i.e., episodic memory, visuospatial function, information processing speed and executive function) and the connectivity values (i.e., FCS and seed-based RSFC) in the areas showing significant between-group differences. First, we found that the FCS values in the right HIP/PHG were negatively correlated with the cognitive performance (i.e., episodic memory and executive function) in the aMCI ε4 carriers (Figure 2, Table 2). Importantly, we further observed that lower HIP/PHG-RSFCs with the bilateral MPFC were positively correlated with the episodic memory and executive function in the aMCI ε4 carriers (Figure 4, Table 4). However, in the aMCI ε3ε3 carriers, the connectivity measures were not significantly correlated with any cognitive performance of episodic memory, visuospatial function information processing speed and executive function (Tables 2, 4). Also, a statistical comparison of the correlations using *Z* statistic further indicated that the association was significantly different between the two groups (Tables 2, 4, all *P*s < 0.05, |Cohen’s *q*| >0.30).

**Table 4:** Correlations between the HIP/PHG-RSFC with the bilateral MPFC and the cognitive performance in aMCI.

	r, APOE ε3ε3 carriers (*n* = 38)	r,APOE ε4+ carriers (*n* = 28)	Fisher’s *Z*	Cohen’s *q*
**MMSE**	**0.128**	0.420*	**−1.2181 (0.1116)**	**−0.3190**
**Episodic memory**	0.253	0.450*	−0.8634 (0.1940)	−0.2261
**Visuospatial function**	0.036	0.163	−0.4905 (0.3119)	−0.1285
**Information processing speed**	0.148	0.189	−1.1612 (0.4360)	−0.0422
**Executive function**	0.194	0.482**	−1.2568 (0.1044)	−0.3291

Multiple linear regression analyses were conducted to calculate the correlations between the HIP/PHG-RSFC with the bilateral MPFC and cognitive performance in the APOE ε3ε3 and APOE ε4 carriers, after controlling for age, gender and education. aMCI, amnestic mild cognitive impairment; APOE, apolipoprotein E; HIP, hippocampus; RSFC, resting-state functional connectivity; MPFC, medial prefrontal cortex; PHG, parahippocampal gyrus *P < 0.05 **P < 0.01. Values in bold means that compared with HC group, these values make significant correlation with HIP/PHG-RSFC in aMCI patients.

## Discussion

Using the resting-state fMRI and graph-theory approaches, we found that the APOE ε4 allele is linked to a specific pattern of the HIP/PHG network in aMCI subjects. The resting-state connectivity disturbance of HIP/PHG-MPFC may be the neurobiological mechanism underlying the cognitive deficits (i.e., episodic memory and executive function) in aMCI ε4 carriers. Also, the HIP/PHG-MPFC may serve as an important predictor of conversion from aMCI to AD.

Defining genetic risk through sensitive functional brain imaging technologies may greatly improve our ability to detect individuals who have the potential to develop into AD in the asymptomatic period. In general, in our present study, an ε4-related increase of FCS in the right HIP/PHG in aMCI patients was first observed (Figure 1 and Supplementary Figure S1). This observation provided evidence in support of the contention that aberrant mesial temporal lobe (MTL) connectivity in the whole brain can be greatly influenced by the genetic risk of AD. The MTL structures are regarded as important neural substrates acting as a pivotal input and output hub in episodic memory, which are always prominent and significantly affected in aMCI and even asymptomatic dementia (Bakker et al., 2012; Kauppi et al., 2014). In AD/aMCI, the hippocampus is thought to be one of the first regions of the brain to suffer damage. Severe amyloid deposition was observed in MTL in both 5XFAD mice (Oh et al., 2018) and patients with AD, using positron emission tomography (PET; Firouzian et al., 2018). The volume of hippocampus (Heise et al., 2014), electroencephalogram coherence of MTL (Jelic et al., 1997), magnetoencephalography resting state functional connectivity in source space in MTL (Cuesta et al., 2015) and functional connectivity in hippocampal networks (Ye et al., 2018) are conspicuous disrupted in subjects of healthy elderly, aMCI and AD with APOE ε4. Numerous studies have found that APOE ε4 can affect the atrophic hippocampal volume and accelerate the atrophy rate in AD and subjects with genetic risks of AD. Also, during AD pathology progression, the MTL formations are hypothesized to show hyperactivation in elders with normal cognitive state at genetic risk of AD (Tran et al., 2017) and aMCI, compared to controls (Jin et al., 2012). It also seems that, 2.5 years later, patients with hyperactivation in MTL had higher AD conversion rates and accelerated worse cognitive decline (Dickerson et al., 2004). This finding suggests that MTL hyperactivation in the aMCI phase may be a predictor for quicker progression to AD. What’s more, the face task-activated fMRI longitudinal studies showed that elders with a normal cognitive state at genetic risk for AD proved a manner of increased MTL activity (compared to those at low risk at baseline). Also, a progressive decrease in activation was shown during the 5-year follow-up periods, which corresponded with the emergence of episodic memory impairment and hippocampal atrophy (Wolk and Dickerson, 2010; Rao et al., 2015). Also, an “inverse U-shaped curve” of hippocampal RSFC in our prior study (Ye et al., 2017) showed the same pattern of activation changes in the hippocampus. Therefore, the activated network within MTL in aMCI ε4 carriers may be an early indicator of AD-related neurodegeneration in a distributed network for cognition reserve, which in turn might result from the pathophysiologic effects of the APOE ε4 allele (Tran et al., 2017).

Subsequently, we made the right HIP/HPG based RSFC maps for further analysis. Notably, compared with the ε3ε3 carriers, the ε4 carriers had lower RSFCs between the right HIP/PHG seed and the bilateral MPFC, and higher RSFCs between the right HIP/PHG seed and the occipital cortex. The MPFC is a region which is considered to play a key role in connecting current episodic processing to prior experience (Bonasia et al., 2018). The MPFC is a major region in DMN, in which the deficiency is always associated with the APOE ε4 allele (Gusnard et al., 2001). The connection between the MPFC and HIP has been implicated in memory transformation (Sekeres et al., 2018). Memory-related increased excitatory neurotransmitter levels in MPFC have also been associated with better memory and stronger memory-related functional connectivity in the HIP-MPFC network (Thielen et al., 2018). Previous fMRI studies have already showed decreased activity in HIP and MPFC, using amplitude of low-frequency fluctuation in aMCI. Further functional connectivity analysis in DMN showed declined functional connectivity between MPFC and HIP (Cai et al., 2017), and this is also shown in episodic memory retrieval processing task (Bai et al., 2009). In addition, ε4 was demonstrated to have significant influence on the MPFC activation in aMCI (Wang et al., 2017a). In the present study, we further found that a lower RSFC between the right HIP/PHG and the MPFC was positively correlated to episodic memory scores in APOE ε4 carriers. Therefore, the impairment of connectivity between HIP and MPFC may be the structural foundation of APOE ε4-related deficiency in episodic memory. In contrast, a higher RSFC between the right HIP/PHG seed and the occipital cortex was observed in aMCI with APOE ε4. Although the occipital cortex is a central visual processing area in the brain (Wandell et al., 2007), this area was also demonstrated to be significantly related to cognitive performance in neurodegenerative disease (Johnson et al., 2015) and emotion recognition (Scahill et al., 2013). Both overload tau and Aβ accumulation were found in the occipital cortex by PET imaging (Cho et al., 2017; Chiotis et al., 2018). Different amplitudes of the occipital sources of low-frequency alpha rhythms (8–10.5 Hz) were shown in aMCI and AD in a resting-state electroencephalographic study (Babiloni et al., 2015). Importantly, the inferior fronto-occipital fasciculus and superior longitudinal fasciculus are long association fiber bundle interconnecting areas within the frontal, parietal, occipital and temporal lobes, which in turn play an important role in a variety of neurological cognitive functions, such as working memory, spatial attention and visual processing. Additionally, greater positive functional connectivity was also observed between the dorsolateral prefrontal cortex (DLPFC) and occipital cortex in AD, using a functional connectivity analysis (De Marco et al., 2017a). Thus, the increased functional connectivity in the occipital cortex may attempt to bolster the stabilization of the functional network in the aMCI ε4 carriers. Furthermore, in terms of cognition performance, no significant differences were detected between ε4 and ε3ε3 carriers. The occipital lobe and MPFC may work cooperatively to sustain the hyperactivation in HIP/PGH for memory reservation. Additionally, the HIP/PHG-RSFC with the bilateral MPFC had an important independent influence on prediction of AD conversion. Importantly, the lower the HIP/PHG-RSFC with the bilateral MPFC was, the higher the conversion rate was. Therefore, FC declining between right HIP/PHG and bilateral MPFC may provide complementary information for the prediction of AD conversion, which makes important sense in the early diagnosis and early prevention of AD. Further longitudinal studies are needed to be conducted to examine whether the network measures could serve as primary predictors of conversion from aMCI ε4 carriers to AD.

Several issues discussed in this study need to be further addressed. First, given that only eight subjects were carrying APOE ε2 allele, they were not involved in further analysis. This limits this study’s efforts in the exploration of APOE genotype effects. Many more aMCI subjects should be recruited in the future, especially those who carry ε2 allele. Second, we exclusively focused on a genetic risk factor of the APOE genotype for aMCI. A considerable biological and clinical heterogeneity (such as amyloid or tau level in cerebrospinal fluid) will be raised in the future. Finally, a tight relationship between the structural and functional connectivity networks in healthy subjects has been documented recently (Hermundstad et al., 2013). Thus, further investigation of APOE E4 effects on the relation between structure and function will be an interesting topic.

## Conclusion

Overall, these findings suggest that the APOE ε4 allele modulates the large-scale brain network in aMCI subjects, thus facilitating our understanding of how the entire brain network assembly reorganizes in response to APOE variants in aMCI. A compensatory recruitment of intact brain regions was revealed. This presumably results from subtle neural dysfunction for cognition reserve, which may be an early signal of incipient disease. Furthermore, our data suggest the combination of altered functional connectivity pattern of HIP/PHG networks and APOE ε4 allele may be a crucial indicator for monitoring the onset and the progression of AD, which is important for early prevention and creating a rational therapy strategy.

## Author Contributions

LZ and ZW are the first authors who mainly analyzed the data and wrote the manuscript. HS and DL made the recruitment of all the subjects. The analysis methods were learnt from QG; and ZZ is the corresponding author.

## Conflict of Interest Statement

The authors declare that the research was conducted in the absence of any commercial or financial relationships that could be construed as a potential conflict of interest.

## Footnotes

^1^http://www.fil.ion.ucl.ac.uk/spm

^2^http://www.restfmri.net/forum/dparsf

^3^http://www.nitrc.org/projects/gretna/

^4^http://afni.nimh.nih.gov/pub/dist/doc/manual/AlphaSim.pdf
