# Supplementary material for: Apolipoprotein E ε4 Specifically Modulates the Hippocampus Functional Connectivity Network in Patients With Amnestic Mild Cognitive Impairment
Source: Front Aging Neurosci. 2018 Sep 27;10:289. doi: 10.3389/fnagi.2018.00289 (PMC6170627; doi:10.3389/fnagi.2018.00289)
Supplement: Supplementary file 1 [file Data_Sheet_1.docx]

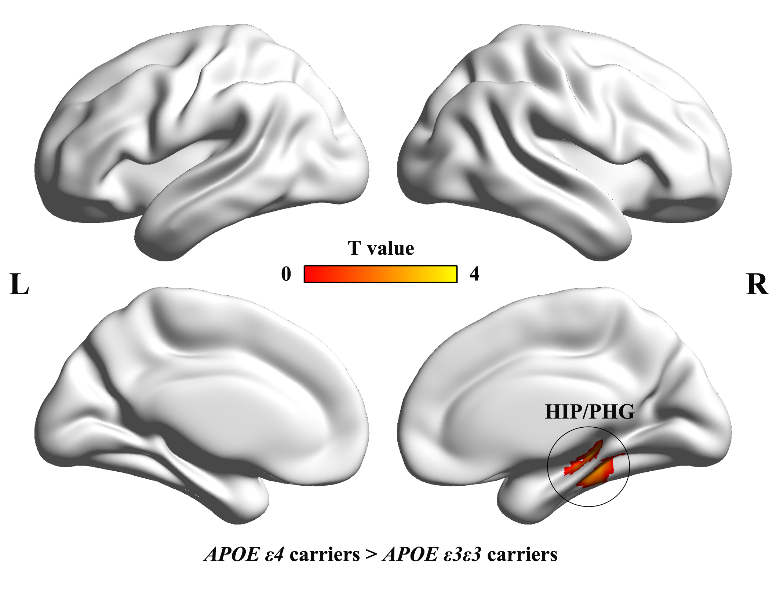


**Supplementary Figure 1: Difference of functional connectivity strength (FCS) map between APOE ε3ε3 carriers and APOE ε4+ carriers corrected by grey matter** Compared with the APOE ε3ε3 carriers, the APOE ε4+ carriers showed significantly increased FCS values in the right hippocampus/parahippocampus. The color bar represents the strength of positive FCS. APOE, apolipoprotein E; HIP, hippocampus; PHG, parahippocampal gyrus

|  | **HC** | | ***P* value** | **Cohen’s *d*** |
| --- | --- | --- | --- | --- |
|  | **APOE ε3ε3** (n=45) | **APOE ε4+** (n=45) |  |  |
| **Percentage female** | 21 (46.67%) | 23 (51.11%) | 0.916 ^a^ | - |
| **Age** (years) | 69.39±5.94 | 68.67±6.00 | 0.303 ^b^ | - |
| **Education level** (category) | 7-17 years | 6-17 years | 0.286 ^b^ | - |
| **General cognition** | | | | |
| MMSE | 28.45±1.13 | 28.09±1.52 | 0.276 ^c^ | 0.27 |
| MDRS-2 total | 137.76±3.54 | 137.76±3.32 | 0.663 ^c^ | 0.00 |
| **Composite Z scores of each cognition domain** | | | | |
| Episodic Memory | 0.01±0.78 | -0.00±0.68 | 0.945 ^c^ | 0.01 |
| AVLT-20min DR | 0.01±0.97 | -0.01±1.94 | 0.968 ^c^ | 0.01 |
| LMT-20min DR | 0.00±1.04 | -0.00±0.97 | 0.805 ^c^ | 0.00 |
| CFT-20min DR | 0.00±1.10 | -0.00±0.90 | 0.897 ^c^ | 0.00 |
| Visuospatial Function | -0.09±0.76 | 0.08±0.70 | 0.243 ^c^ | -0.01 |
| CDT | -0.04±0.99 | 0.04±1.02 | 0.658 ^c^ | -0.08 |
| CFT | -0.13±1.06 | 0.13±0.93 | 0.210 ^c^ | -0.26 |
| Information Processing Speed | -0.00±0.74 | 0.00±0.85 | 0.743 ^c^ | 0.00 |
| DSST | -0.02±0.99 | 0.02±1.02 | 0.618 ^c^ | -0.04 |
| TMT-A | -0.04±0.96 | 0.04±1.05 | 0.622 ^c^ | -0.08 |
| Stroop A | -0.04±0.87 | 0.04±1.12 | 0.504 ^c^ | -0.08 |
| Stroop B | 0.09±0.94 | -0.08±1.06 | 0.509 ^c^ | 0.17 |
| Executive Function | 0.01±0.60 | -0.01±0.73 | 0.926 ^c^ | 0.03 |
| VFT-objects | 0.10±0.97 | -0.09±1.03 | 0.354 ^c^ | 0.19 |
| VFT-animals | 0.14±1.01 | -0.14±0.98 | 0.317 ^c^ | 0.28 |
| DST-backward | -0.17±0.95 | 0.17±1.03 | 0.113 ^c^ | -0.34 |
| TMT-B | 0.01±0.95 | -0.01±1.05 | 0.953 ^c^ | 0.02 |
| Stroop C | 0.06±0.88 | -0.06±1.11 | 0.528 ^c^ | 0.12 |
| Similarity | -0.07±1.07 | 0.07±0.93 | 0.322 ^c^ | -0.14 |

**Supplementary Figure 2 (only for reviewer):** Data are presented as mean ± standard deviation. The level of each cognitive domain and performance of each neuropsychological test are expressed as Z scores. HC, healthy control; APOE, apolipoprotein E; MMSE, mini-mental state exam; MDRS-2, mattis dementia rating scale-2. AVLT, auditory verbal learning test; LMT: logical memory test; CFT: Rey-Osterrieth complex figure test; CDT, clock drawing test; DSST, digital symbol substitution test; TMT-A, trail making test-A; Stroop, stroop color test; VFT, verbal fluency test; DST, digit span test; TMT-B, trail making test-B; Similarity, semantic similarity test. ^a^ *P* values were obtained by χ^2^ tests. ^b^ *P* values were obtained by independent samples t-test. ^c^ *P* values were obtained by one-way analysis of covariance (ANCOVA)
